# Proposing a Sex-Adjusted Sodium-Adjusted MELD Score for Liver Transplant Allocation

**DOI:** 10.1001/jamasurg.2022.1548

**Published:** 2022-05-18

**Authors:** Julia M. Sealock, Ioannis A. Ziogas, Zhiguo Zhao, Fei Ye, Sophoclis P. Alexopoulos, Lea Matsuoka, Guanhua Chen, Lea K. Davis

**Affiliations:** 1 Division of Hepatobiliary Surgery and Liver Transplantation, Vanderbilt University Medical Center, Nashville, Tennessee; 2 Department of Biostatistics, Vanderbilt University Medical Center, Nashville, Tennessee; 3 Department of Biostatistics and Medical Informatics, University of Wisconsin-Madison, Madison; 4 Division of Genetic Medicine, Department of Medicine, Vanderbilt University Medical Center, Nashville, Tennessee; 5 Vanderbilt Genetics Institute, Vanderbilt University Medical Center, Nashville, Tennessee; 6 Department of Molecular Physiology and Biophysics, Vanderbilt University Medical Center, Nashville, Tennessee; 7 Department of Psychiatry and Behavioral Sciences, Vanderbilt University Medical Center, Nashville, Tennessee; 8 Department of Medicine and Biomedical Informatics, Vanderbilt University Medical Center, Nashville, Tennessee

## Abstract

**Importance:**

Liver allocation is determined by the model for end-stage liver disease (MELD), a scoring system based on 4 laboratory measurements. During the MELD era, sex disparities in liver transplant have increased and there are no modifications to MELD based on sex.

**Objective:**

To use laboratory values stored in electronic health records to describe population-level sex differences in all MELD laboratory values (in healthy individuals and patients with liver disease) and propose a sex adjustment.

**Design, Setting, and Participants:**

A retrospective cohort study was conducted from March 2019 to April 2020 to evaluate sex differences in laboratory values in liver transplant patients, patients with liver disease who did not undergo transplant, and healthy controls. Primary analyses were conducted in Vanderbilt University Medical Center (VUMC)’s deidentified electronic health record system. Replication analyses were conducted in the All of Us Research Program. Simulations of a sex-adjusted sodium-adjusted MELD (MELDNa) score were completed using liver transplant waiting list data from the liver simulated allocation modeling system. Patients who regularly used VUMC with measurements for any MELDNa component laboratory were included in the analyses. Analysis took place from November 2019 to March 2021.

**Exposures:**

Electronic health record–reported sex.

**Main Outcomes and Measure:**

Creatinine, bilirubin, international normalized ratio, and sodium levels.

**Results:**

The VUMC sample was composed of 623 931 individuals (359 976 [57.7%] female) with a median (IQR) age of 44 (23-61) years. All component MELDNa laboratory values and calculated MELDNa scores yielded significant sex differences within VUMC (mean [SD] creatinine: male, 0.99 [0.39] mg/dL; female, 0.79 [0.30] mg/dL; *P* < .001; bilirubin: male, 0.76 [0.83] mg/dL; female, 0.58 [0.64] mg/dL; *P* < .001; international normalized ratio of prothrombin rate: male, 1.24 [0.42]; female, 1.20 [0.40]; *P* < .001; sodium: male, 139.00 [2.36] mEq/L; female, 139.03 [2.28] mEq/L; *P* < .001), resulting in MELDNa scoring that disadvantaged female individuals. This pattern persisted when the sample was divided into healthy controls, individuals with liver disease who did not undergo transplant, and patients who did undergo liver transplant. Female transplant patients had a greater number of decompensation traits (mean [SD]: male, 1.34 [1.11]; female, 1.60 [1.09]; *P* = .005), despite having lower MELDNa scores (mean [SD]: male, 21.72 [6.11]; female, 20.21 [6.15]; *P* = .005), indicating MELDNa scores are not accurately representing disease severity in female individuals. In simulations, the sex-adjusted MELDNa score modestly increased female transplant rate and decreased overall death.

**Conclusions and Relevance:**

These results demonstrate pervasive sex differences in all laboratory values used in MELDNa scoring and highlight the need and utility of a sex-adjustment to the MELDNa protocol.

## Key Points

**Question:**

Do sex differences in sodium-adjusted model for end-stage liver disease (MELDNa) laboratory values persist in a hospital population, and can electronic health records be used to adjust for sex in MELDNa scoring?

**Findings:**

In this cohort study of 623 931 individuals, using laboratory values extracted from electronic health records, all MELDNa component laboratory values showed significant sex differences that disadvantaged female individuals, even in individuals without evidence of liver disease. A sex-adjusted MELDNa score simulated in liver transplant waiting list data increased female transplant rate and decreased overall death.

**Meaning:**

Pervasive sex differences exist in all laboratory values used in MELDNa scoring and highlight the need and utility of a sex adjustment to the MELDNa protocol.

## Teaser

This cohort study uses laboratory values stored in electronic health records to describe population-level sex differences in all model for end-stage liver disease (MELD) laboratory values (in healthy controls and individuals with liver disease) and propose a sex adjustment.

## Introduction

In 2002, the Organ Procurement and Transplantation Network (OPTN) adopted the model for end-stage liver disease (MELD) scoring system to allocate livers based on objective medical criteria. MELD scores were initially calculated from 3 laboratory values: creatinine, international normalized ratio of prothrombin rate (INR), and bilirubin.^1^ The MELD allocation system is based on the “sickest first” principle whereby individuals with the highest scores have priority access to organs. In some cases, MELD does not adequately capture the severity of illness, such as hepatocellular carcinoma, hepatopulmonary syndrome, and portopulmonary hypertension.^2^ Individuals with these diagnoses receive exception points to increase their listing MELD scores and decrease their time to transplant. Additionally, other factors can affect MELD’s mortality prediction,^2^ including hyponatremia, nutritional status, and sex.^3,4^ These effects on MELD’s prediction led to several proposed modifications to MELD scoring,^2^ including the sodium-adjusted MELD score (MELDNa),^5,6^ which has replaced the original MELD in clinical practice. Notably, sex disparities in liver transplant have widened in the MELD era^7,8^; however, no modifications to MELD based on sex have been accepted.

Sex differences in liver transplant are well documented. Female individuals spend longer on the waiting list,^8,9^ are more likely to die or become too sick for transplant while on the waiting list,^3,8,10,11,12^ and are less likely to receive MELD exception points than male individuals.^13^ Previous studies attempted to explain the sex disparity in liver transplants by investigating factors such as size mismatches between donor-recipient pairs, geographic disparities, and lower creatinine levels in female individuals. After controlling for estimated liver size^12,14^ or height,^15^ sex disparities in transplant were reduced but not eliminated. Likewise, controlling for geographic disparities did not ameliorate the sex disparity in receiving a liver transplant.^12^ Several studies postulated that decreased body size in female individuals leads to lower creatinine levels, which in turn disadvantages female individuals in MELD scoring.^3,16,17^ Indeed, male individuals tend to be listed with higher creatinine, estimated glomerular filtration rate (eGFR), and MELD scores than female individuals,^3,4,16,17,18^ but replacement of creatinine with eGFR did not improve the MELD model in female individuals.^3,11,18^ The lack of explanation from previous studies suggests no single factor can fully explain the sex difference in liver transplants, but rather a constellation of differences exist between male and female individuals, leading to the observed disparity.

The majority of previous studies investigating sex differences in liver transplant used data from the United Network for Organ Sharing database, which allows for analysis of demographics, cause of liver disease, MELD scores at listing, comorbidities, and outcomes. However, the United Network for Organ Sharing database does not allow for investigation of sex differences at the population level. Electronic health records (EHRs) store longitudinal information on the health and clinical care of individuals, including diagnoses, procedures, medications, and laboratory test results. Rather than conducting studies in traditionally collected cohorts, EHRs enable research on the entire population of a health care system, which can increase sample size, reduce bias based on ascertainment, and increase generalizability. In this study, we leverage EHR data from a single tertiary care medical center and a multisite initiative, the All of Us Research Program,^19^ to investigate sex differences in the (1) laboratory traits composing MELDNa scores, (2) calculated MELDNa scores, and (3) number of liver decompensation traits. To form a complete picture of the scope of sex differences affecting MELD, we included a range of clinical end points from healthy controls, individuals with liver disease, and individuals who underwent transplant. Finally, we derive a sex-adjusted MELDNa score and test its ability to reduce sex disparities in simulated liver transplant waiting list data to ensure applicability to a transplant-specific sample.

## Methods

### Data Source

Vanderbilt University Medical Center (VUMC) is a tertiary care center that provides inpatient and outpatient care in Nashville, Tennessee. The VUMC EHR was established in 1990 and includes data on billing codes from the *International Classification of Diseases, Ninth Revision (ICD-9) *and* Tenth Revision (ICD-10)*, *Current Procedural Terminology* (*CPT*) codes, laboratory values, reports, and clinical documentation. The deidentified mirror of the EHR numbers more than 3.2 million patient records. This study was deemed nonhuman subjects research by the VUMC institutional review board (IRB#172020).

### Study Sample

To enrich our sample for individuals who regularly use VUMC for their primary care, we implemented a data floor heuristic (at least 5 *ICD* codes received throughout a period of at least 3 years). Because liver cancer and kidney dialysis affect MELDNa scores, individuals with the presence of the liver cancer *ICD* codes (155-155.2, C22-C22.9) or dialysis *CPT* codes (90935-90999) were excluded (eTable 1 in Supplement 1). Liver transplant cases were defined by the presence of the liver transplant *CPT* code, 47135 (n = 601). The remaining sample was further stratified by liver disease status, determined by the presence of at least 2 component chronic liver disease *ICD* codes (Supplement 2), indicative of individuals who might qualify for a transplant but have not yet received one (n = 24 921). Individuals with only 1 liver disease code were excluded to reduce potential false-positive cases. The remaining sample was classified as healthy controls. In this study, healthy control status refers to the absence of liver disease. EHRs are not linked to the liver transplant waiting list system; therefore, we were not able to use waiting list–specific information, such as listing date, dual listing status, or delisting status. Race is collected as an EHR-reported variable, but it was not included in the analysis because it is not part of the MELD calculation, nor does it otherwise explicitly factor into the transplant allocation.

### Statistical Analysis

#### Laboratory Values

MELDNa component laboratory values (creatinine, INR, bilirubin, and sodium) were extracted from the EHR and filtered for observations more than 8 SDs from the group sample mean, indicative of data entry error or biologically implausible values. Given the longitudinal nature of the EHR, we selected the median and maximum of each laboratory value per individual. In individuals who underwent liver transplant, we selected the median and maximum laboratory values prior to transplant, as defined as the date of liver transplant *CPT* code (47135). In individuals with liver disease and in controls, the median and maximum laboratory values across the entire medical record were selected (eTable 2 in Supplement 1). In primary analyses, we applied *t* tests to determine the differences in group means of the median laboratory values between male and female individuals within the entire sample, within healthy controls, within the liver disease sample, and within the liver transplant sample. In sensitivity analyses, sex differences in median laboratory values were assessed using Wilcoxon rank sum tests to account for non-normality and analysis of covariance tests controlling for decompensation count to account for disease severity. Sex differences in maximum laboratory values were tested in sensitivity analyses using *t* tests (eTable 8 and eFigure 1 in Supplement 1).

#### OPTN MELDNa Scores

OPTN MELDNa scores were calculated using all available 8-SD filtered creatinine, INR, bilirubin, and sodium values. To most closely estimate the clinically relevant scores, we required that all 4 laboratory values contributing to MELDNa score calculations were recorded on the same date. For individuals with liver disease and healthy controls, median and maximum OPTN MELDNa scores across the entire medical record were used for subsequent analyses. For liver transplant cases, median and maximum OPTN MELDNa scores prior to liver transplant *CPT* code were used to more closely estimate pretransplant liver disease severity. Differences in group means of OPTN MELDNa scores were assessed between male and female individuals across the entire sample and within liver transplant, liver disease, and control definitions, using *t* tests in primary analyses. In sensitivity analyses, sex differences in MELDNa values were assessed using Wilcoxon rank sum tests to account for non-normality and analysis of covariance tests controlling for decompensation count to account for disease severity (eTable 6 in Supplement 1).

#### Decompensation Measurements

Decompensation traits were defined using *ICD-9* and *ICD-10* codes for hematemesis, gastrointestinal hemorrhage, ascites, jaundice, and hepatic encephalopathy (eTable 3 in Supplement 1). We required individuals to have at least 2 instances of a component code to be coded with any one of the compensation traits. In individuals with liver transplant, only *ICD* codes prior to the liver transplant *CPT* date were used. Decompensation counts were then determined by summing the number of coded decompensation traits for each individual (minimum, 0; maximum, 5) (eTable 4 in Supplement 1). Differences in group means of mean decompensation counts were assessed between male and female individuals across the entire sample and within liver transplant, liver disease, and control definitions, using *t* tests. To account for non-normality, sex differences in decompensation counts were also assessed using Wilcoxon rank sum tests (eTable 9 in Supplement 1).

#### Replication in the All of Us Research Program

We replicated our findings in the All of Us Research Program, a population-based cohort that contains demographic, EHR, and survey information on participants. Even though the sample size of patients who underwent transplant is small in All of Us (n = 25), the broader sample allowed us to confirm the pattern of sex differences in MELDNa laboratory values and further demonstrate pervasive sex differences even in the absence of liver transplant or disease. Detailed methods and results are in the eAppendix and eTables 5 and 7 in Supplement 1.

#### Development of Sex-Adjusted MELDNa Map

Individuals in VUMC with evidence of liver disease but who had not undergone a liver transplant were used to develop a sex-adjusted MELDNa score map. A fair MELDNa score should satisfy the property that a male and a female individual who have similar scores should also have similar liver disease severity. However, as shown in previous literature and corroborated by our work, current OPTN MELDNa score underestimates the degree of illness in female individuals with liver disease. To reduce this disparity, our mapping focused on creation of a score such that mapped sex-adjusted scores resulted in more similar distributions of disease severity for male and female individuals. We created the sex-adjusted MELDNa map with the following 3 steps. First, female and male individuals were matched on race, age at MELDNa score, and decompensation count, which we used as the surrogate for the liver disease severity using nearest-neighbor matching in the MatchIt package (R Foundation).^20^ We divided the matched sample by sex and separately calculated quantiles of MELDNa scores. Because liver transplant waiting list listing dates are not available in EHRs, we used the first calculated MELDNa score after liver disease diagnosis to most closely estimate a patient’s listing MELDNa score on the transplant waiting list. Corresponding quantiles were matched to produce the final map. The quantile mapping reduces the underestimation bias of MELDNa scores for female individuals and preserves the ranking of OPTN MELDNa scores within female individuals. In other words, considering 2 female individuals, the female individual who has a higher OPTN MELDNa score than the other would still have a larger (or equal) sex-adjusted MELD score. Additionally, this approach accommodates nonlinearity in sex-differences, which can result in a smaller sex difference in outcomes for the extreme high MELDNa scores.

#### Evaluating Sex-Adjusted MELDNa Scores in Liver Simulated Allocation Modeling 

Because the sex-adjusted score was derived using EHR data, which may include waitlisted and nonwaitlisted patients, we next sought to validate the score in a liver transplant waiting list sample. Liver simulated allocation modeling (LSAM) is a discrete event simulation that models the functioning of the US liver allocation system and allows for comparison of various allocation schemes.^21^ The LSAM software was used to model outcomes of changing OPTN MELDNa scores to the proposed sex-adjusted scores. Simulations using both the OPTN MELDNa score and the proposed sex-adjusted MELDNa score were conducted with 10 replications of organ and waiting list arrivals from most recent liver transplant data (2015.6-2016.6) contained in the LSAM package. Within each replication, the same simulated data set was used for both MELDNa scores to generate comparative estimates of transplant rate, waiting list mortality, and the impact of these with regard to sex disparity in receiving liver transplant. Reported results are the means across each replication. The primary outcome of interest is the impact of the sex-adjusted MELDNa on sex-specific total transplant rate compared with the current MELDNa. Secondary outcomes included 1-year waiting list mortality (per 100 person-years) and total deaths.

All analyses were completed in R version 3.4 or 4.0.0 (R Foundation). A Bonferroni correction was used to account for multiple testing across the VUMC sample; significance was set at *P* < .008. All *P* values were 2-sided.

## Results

### Sex Differences in Component Laboratory Values and Calculated MELDNa Scores

The VUMC sample was composed of 623 931 individuals (359 976 [57.7%] female) with a median (IQR) age of 44 (23-61) years. Across all individuals, all component MELDNa lab values yielded significant sex differences within VUMC (mean [SD] creatinine: male, 0.99 [0.39] mg/dL; female, 0.79 [0.30] mg/dL; *P* < .001; bilirubin: male, 0.76 [0.83] mg/dL; female, 0.58 [0.64] mg/dL; *P* < .001; international normalized ratio of prothrombin rate: male, 1.24 [0.42]; female, 1.20 [0.40]; *P* < .001; sodium: male, 139.00 [2.36] mEq/L; female, 139.03 [2.28] mEq/L; *P* < .001).

**Figure 1.**
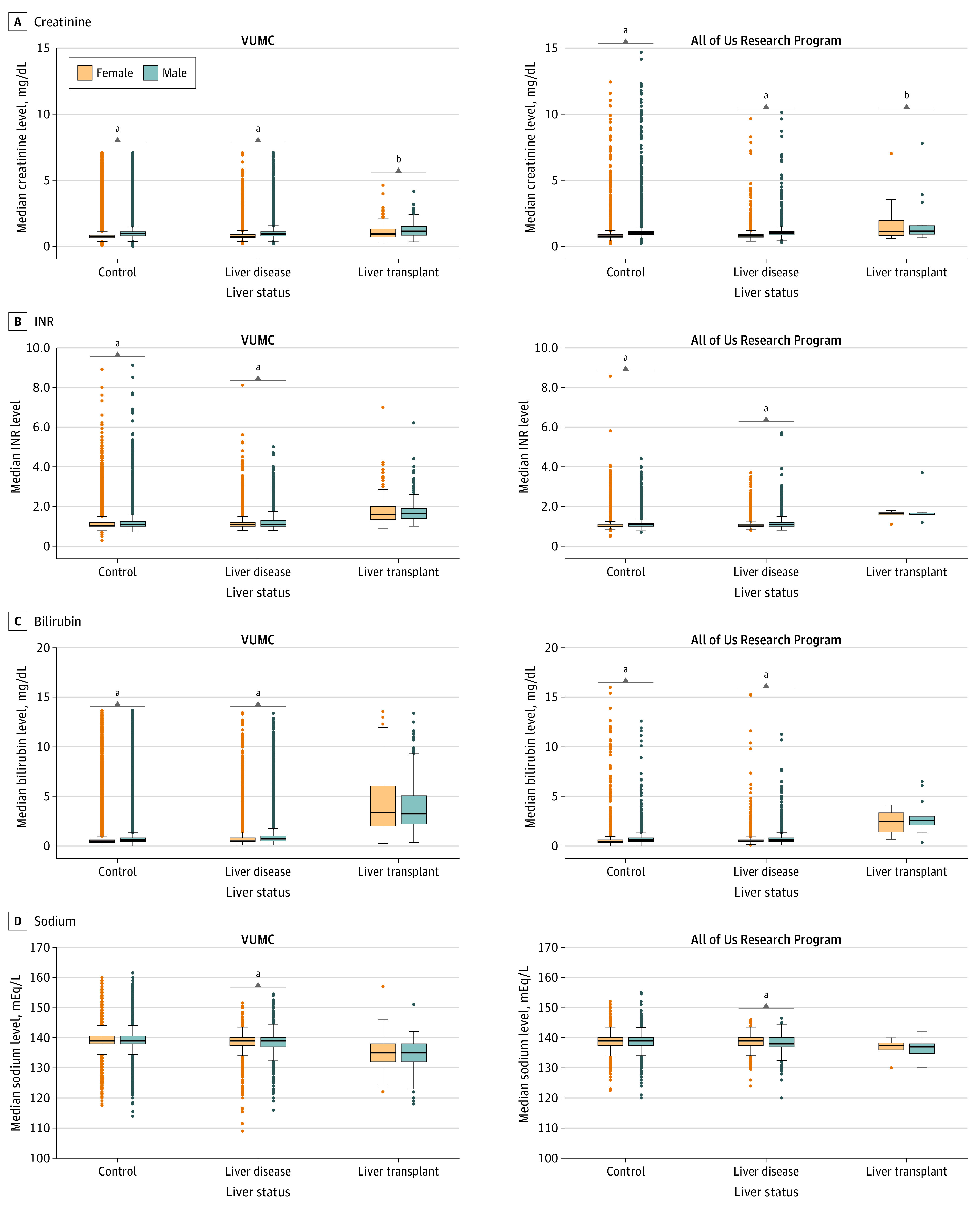
Sex Differences in Model for End-Stage Liver Disease Laboratory Values Sex differences in median creatinine (A), international normalized ratio of prothrombin rate (INR) (B), bilirubin (C), and sodium (D) levels stratified by liver status in Vanderbilt University Medical Center (VUMC) and the All of Us Research Program. For liver transplant recipients, median levels prior to liver transplant date were used. For controls and individuals with liver disease, median levels across the entire electronic health record were used. Sex differences were assessed using a *t* test. ^a^*P* < .001. ^b^*P* ≤ .01.

There were 598 409 healthy controls. Within controls, compared with female individuals, male individuals had increased mean (SD) levels of creatinine (0.99 [0.39] mg/dL vs 0.79 [0.29] mg/dL [to convert to micromoles per liter, multiply by 88.4]; *P* < .001), INR (1.24 [0.42] vs 1.19 [0.40]; *P* < .001), and bilirubin levels (0.73 [0.77] mg/dL vs 0.56 [0.59] mg/dL [to convert to micromoles per liter, multiply by 17.104]; *P* < .001), but sodium levels were not different (139.06 [2.32] mEq/L vs 139.06 [2.26] mEq/L [to convert to micromoles per liter, multiply by 1]; *P* = .60). Male individuals with liver disease showed increased mean (SD) levels of creatinine (1.02 [0.46] mg/dL vs 0.83 [0.63] mg/dL; *P* < .001), INR (1.24 [0.37] vs 1.20 [0.36]; *P* < .001), and bilirubin levels (0.99 [1.11] mg/dL vs 0.78 [1.00] mg/dL; *P* < .001), as well as decreased sodium levels (138.34 [2.64] mEq/L vs 138.65 [2.39] mEq/L; *P* < .001) compared with female individuals. In individuals with liver transplant, male individuals had increased mean (SD) pretransplant creatinine levels compared with female individuals (1.26 [0.55] mg/dL vs 1.11 [0.57] mg/dL; *P* = .001), but pretransplant INR (1.78 [0.59] vs 1.80 [0.73]; *P* = .74), bilirubin (4.00 [2.57] mg/dL vs 4.27 [3001] mg/dL; *P* = .26), and sodium levels (134.46 [4.69] mEq/L vs 134.80 [4.34] mEq/L; *P* = .36) were not significantly different (Figure 1A-D; eTable 6 in Supplement 1).

**Figure 2.**
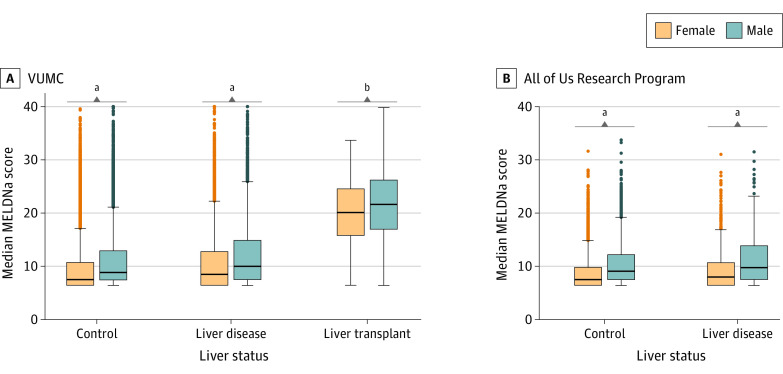
Sex Differences in Median Calculated Organ Procurement and Transplantation Network Model for End-Stage Liver Disease (MELDNa) Scores Stratified by Liver Status in Vanderbilt University Medical Center (VUMC) and the All of Us Research Program For liver transplant recipients, median levels prior to liver transplant date were used. For controls and individuals with liver disease, median levels across the entire electronic health record were used. Sex differences were assessed using a *t* test. ^a^*P* < .001. ^b^*P* ≤ .01.

Across all individuals, calculated MELDNa scores were higher among male than female individuals in VUMC (mean [SD] score: male, 11.09 [5.19]; female, 9.78 [4.70]; *P* < .001). Mean (SD) MELDNa scores were increased in male individuals compared with female individuals within each liver status group (controls: 10.80 [4.93] vs 9.54 [4.44]; *P_ _*<*_ _*.001; individuals with liver disease: 12.01 [5.77] vs 10.68 [5.40]; *P_ _*<*_ _*.001; individuals who underwent liver transplant: 21.72 [6.11] vs 20.21 [6.15]; *P = *.005) (Figure 2; eTable 6 in Supplement 1).

### Sex Differences in Decompensation Counts

Among controls and individuals with liver disease, male individuals had higher mean (SD) decompensation counts than female individuals (controls: 0.14 [0.13] vs 0.01 [0.11]; *P_ _*<*_ _*.001; individuals with liver disease: 0.25 [0.61] vs 0.21 [0.56]; *P_ _*<*_ _*.001). However, among patients who had received a transplant, female individuals had higher mean (SD) pretransplant decompensation counts (1.34 [1.11] vs 1.60 [1.09]; *P* = .005) (eTable 9 and eFigures 2 and 3 in Supplement 1). The difference in the mean number of decompensation traits between individuals with liver disease and those who underwent liver transplant was 1.4 for female individuals and 1.1 for male individuals, suggesting female individuals generally accrue more decompensation traits prior to receiving a liver transplant.

### Development of Sex-Adjusted MELDNa Map

**Figure 3.**
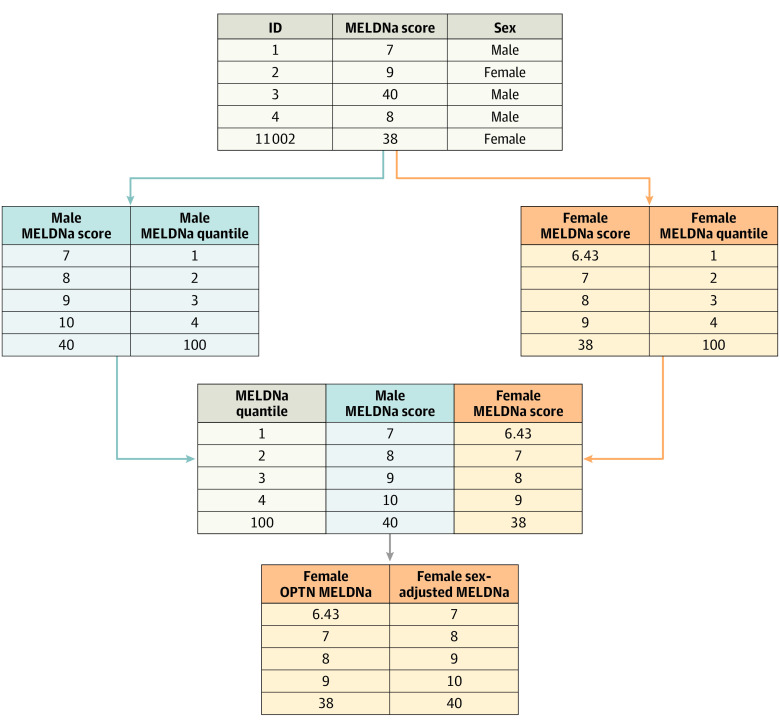
Derivation of the Sex-Adjusted Sodium-Adjusted Model for End-Stage Liver Disease (MELDNa) Map In Vanderbilt University Medical Center, patients with liver disease who did not undergo transplant were matched by sex on number of decompensation traits, age at MELDNa score, and race. Next, quantiles of MELDNa scores were calculated separately for male and female individuals. MELDNa scores were then matched based on quantile. The sex-adjusted map converts the original female Organ Procurement and Transplantation Network (OPTN) score to the corresponding male score.

Male MELDNa scores remain based on the OPTN calculation while the female sex-adjusted MELDNa score is found in the sex-adjusted MELDNa column corresponding to the unadjusted OPTN MELDNa score (Table 1 and Figure 3). Female scores were increased between a mean of 0 to 2 points and 1.6 points.

**Table 1.** Proposed Sex-Adjusted MELDNa Score Map^a^

Female MELDNa score	
OPTN	Sex-adjusted
6	7
7	9
8	9
9	11
10	12
11	13
12	14
13	15
14	16
15	17
16	18
17	19
18	20
19	21
20	22
21	23
22	24
23	25
24	26
25	27
26	28
27	28
28	29
29	30
30	32
31	33
32	34
33	35
34	36
35	36
36	37
37	37
38	38
39	39
40	40

Abbreviations: MELDNa, sodium-adjusted model for end-stage liver disease; OPTN, Organ Procurement and Transplantation Network.

^a^ Female sex-adjusted MELDNa scores are found in the sex-adjusted column corresponding to the unadjusted OPTN MELDNa score. Male MELDNa scores remain based on the OPTN calculation.

### Evaluating Sex-Adjusted MELDNa Scores in LSAM

Using the current OPTN MELDNa scores, male individuals underwent transplant at a 0.7% higher rate than female individuals (male individuals, 3999 of 16 855 [23.7%]; female individuals, 2344 of 10 177 [23.0%]). When the sex-adjusted scores were assessed, female individuals received transplant at a 1% higher rate than male individuals (male individuals, 3894 of 16 855 [23.1%]; female individuals, 2451 of 10 177 [24.1%]). Total death counts for both sexes decreased using the sex-adjusted scores and waiting list death rates decreased among female individuals (OPTN, 747 of 5127 [15%]; sex-adjusted, 731 of 5098 [14%]) while remaining unchanged in male individuals (OPTN: 1373 of 8453 [16%]; sex-adjusted: 1383 of 8483 [16%]) (Table 2).

**Table 2.** Summary of Liver Simulated Allocation Modeling Simulation Results Comparing OPTN With Sex-Adjusted MELDNa Scores^a^

MELDNa version	Sex	Participants, No.	Transplant (proportion), No. (%)	Total 1-y death	Waiting list death, No. (%)
OPTN	All	27 032	6343 (23.5)	2493	2120 (16)
	Male	16 855	3999 (23.7)	1613	1373 (16)
	Female	10 177	2344 (23.0)	880	747 (15)
Sex-adjusted	All	27 032	6345 (23.5)	2480	2114 (16)
	Male	16 855	3894 (23.1)	1608	1383 (16)
	Female	10 177	2451 (24.1)	872	731 (14)

Abbreviations: MELDNa, sodium-adjusted model for end-stage liver disease; OPTN, Organ Procurement and Transplantation Network.

^a^ Results are calculated as mean values across 10 iterations using independent sets of organ and waiting list arrivals from June 2015 to June 2016.

## Discussion

The MELD allocation system is designed to treat each individual equally; however, equality does not always ensure equity. Uncharacterized differences between groups in clinical laboratory values can exacerbate health disparities. Previous studies ascribed sex differences in MELDNa scores to known sex differences in creatinine levels,^3,16,17,18^ but creatinine does not fully account for the sex difference in MELDNa scores.^3,11,18^ Using 2 EHR systems, we analyzed sex differences in all component laboratory values of MELDNa at scale and in diverse clinical presentations (ie, controls, individuals with liver disease, and individuals who underwent transplant). Our analyses showed significant sex differences in all 4 component laboratory values of MELDNa, which systematically disadvantaged female individuals. Even though the differences were small, the use of a natural logarithm of each laboratory value in the MELDNa calculation magnifies the differences, resulting in increased baseline MELDNa scores in male vs female individuals.

Female individuals are more likely to die or be delisted owing to disease severity while on the liver transplant waiting list.^8,10,12^ Concordantly, in our sample, female individuals who had undergone a liver transplant had significantly more pretransplant decompensation traits than male individuals. However, among individuals with liver disease who had not undergone a transplant, female individuals had fewer decompensation traits. This pattern of results suggests that by the time female individuals qualify for liver transplant, they are, on average, sicker than male individuals, consistent with previous reports of higher waiting list mortality or removal for female individuals.^3,4,8,10^ Furthermore, female liver transplant recipients had lower MELDNa scores despite having more decompensation traits than male transplant recipients, demonstrating that OPTN MELDNa scores may not accurately reflect liver disease progression in female individuals. Although not examined in this study, previous studies confirmed height and liver size contribute to the sex disparity in liver transplant. However, even after controlling for height or estimated liver size, rates of transplant remained lower among female individuals.^4,14,22^

The use of EHRs provides a unique view of sex difference in MELDNa scoring. Whereas previous studies were limited to only individuals with advanced liver disease, we were able to examine sex differences across multiple clinical end points. Additionally, we investigated sex differences in all MELDNa component laboratory values, rather than restricting to only creatinine. Finally, the use of *ICD* codes enabled us to determine degree of liver decompensation to provide another metric of disease severity apart from MELDNa scoring.

We proposed and tested a sex-adjusted MELDNa score map to compensate for the sex differences seen in MELDNa component laboratory values. The sex-adjusted score empirically matches a female individual’s MELDNa score to a male individual’s score of equal illness as defined by an individual’s number of decompensation traits. The majority of sex-adjusted scores for female individuals increase 1 or 2 points from the corresponding OPTN MELDNa score, consistent with a previous report of increased liver transplant access for female individuals with an additional 2 points added to their MELD scores.^15^ While a blanket addition of 1 to 2 points may overcorrect scores for female individuals, especially at higher scores, our proposed adjustment ameliorates this issue by providing a score anchored to disease severity (ie, decompensation trait count). When compared with a recently proposed sex-adjusted MELDNa score, MELDNa-Shift,^23^ our mapping differs by a mean of 1 point, suggesting our models are converging on a common result.

The LSAM models using the sex-adjusted scores showed an increase in female transplant rate and a decrease in overall death for both sexes, suggesting modest but positive improvement in reducing the sex disparity. Using the sex-adjusted scores, female individuals had a 1% higher transplant rate (compared with a 0.7% higher rate for male individuals using the OPTN scores). While this adjustment resulted in a slight increase in female transplant rates compared with male individuals, it is not possible to determine if this is due to an overcorrection for female scores or if female individuals on the waiting list had more severe disease that warranted a higher transplant rate. Additionally, current LSAM accept/decline models do not take into account height, leading to a underestimation of the sex disparity in transplant rates.^23^ A future incorporation of height into LSAM might result in equal rates of transplant between male and female individuals using our sex-adjusted score. Regardless, total death counts were reduced for both male and female individuals and reduced female waiting list mortality, suggested that the sex-adjusted scores could help save lives.

### Limitations

Analysis of EHR data comes with several limitations. EHRs are not linked to national databases, such as the liver transplant waiting list, preventing us from integrating listing and delisting dates into our analyses. Additionally, clinical laboratory tests are not ordered uniformly, potentially creating diagnostic bias in our sample, especially in controls without liver disease. Currently, the All of Us Research Program has a limited number of liver transplant recipients, which limited our ability to assess sex differences in this group. However, all other associations tested replicated in the All of Us data.

Previous studies have shown race-based differences in laboratory values, particularly kidney-related markers such as creatinine and eGFR^24,25^ in which Black individuals have higher values than White individuals. There is a growing recognition that race, a socially derived construct, is not appropriate for stratification of patient populations, and an ongoing examination of the impact of replacing race with genetic ancestry.^26,27^ For example, differences in eGFR persist when race is replaced with genetic ancestry, suggesting that population-differentiated allele frequencies contribute, in part, to differences in laboratory values.^24,28,29^ Consistent with current practice, our analyses did not take into consideration the effects of race or genetic ancestry on MELDNa component laboratory values. However, we believe future modifications of MELDNa should investigate the effects of genetic ancestry on component laboratory values as well as the potential interaction effects between genetic ancestry and sex.

The proposed sex-adjusted MELDNa score does not solve other known causes of sex disparities including donor-recipient size mismatch or geographic or racial disparities. To successfully eliminate the sex disparity in liver transplant, further investigation and potential policy changes, such as access to pediatric donors for female recipients, increase in use of partial liver transplant for female recipients, and increase sharing of donors across United Network for Organ Sharing regions, are necessary.

## Conclusions

Using EHR data, we demonstrate all laboratory traits used in the calculation of MELDNa scores show sex differences that increase male individuals’ scores compared with female individuals’, despite female individuals showing greater liver decompensation. In simulations, our proposed sex-adjusted MELDNa score increases the rate of transplant for female individuals and decreases overall death in both sexes.

## Notes

Supplement 1. eAppendix. eTable 1. Sample characteristics of Vanderbilt University Medical Center Synthetic Derivative (VUMC) and All of Us eTable 2. Descriptive statistics of median MELDNa component labs and median calculated MELDNa stratified by sex and liver status in VUMC eTable 3. Decompensation phenotype ICD9 and ICD10 codes eTable 4. Number of individuals with decompensation traits stratified by sex and liver status eTable 5. Descriptive statistics of MELDNa component labs in the All of Us Research Program eTable 6. Sex differences in median MELDNa component labs and calculated MELDNa stratified by liver status in VUMC eTable 7. Sex differences in median MELDNa component labs and calculated MELDNa stratified by liver status in All of Us eTable 8. Sex differences in maximum MELDNa component labs and calculated MELDNa stratified by liver status in VUMC eTable 9. Sex differences in average decompensation counts stratified by sex and liver status eFigure 1. Sex differences in maximum a) creatinine, b) INR, c) bilirubin, d) sodium, and e) calculated MELDNa stratified by liver status in VUMC eFigure 2. Sex differences in decompensation counts stratified by liver status in VUMC and All of Us eFigure 3. Decompensation counts by median calculated MELDNa stratified by sex and liver status

Supplement 2. ICD-9 and ICD-10 codes used to define liver disease cases

## References

[1] Wiesner R, Edwards E, Freeman R, ; United Network for Organ Sharing Liver Disease Severity Score Committee. Model for end-stage liver disease (MELD) and allocation of donor livers. Gastroenterology. 2003;124(1):91-96. doi:10.1053/gast.2003.50016 12512033

[2] Bernardi M, Gitto S, Biselli M. The MELD score in patients awaiting liver transplant: strengths and weaknesses. J Hepatol. 2011;54(6):1297-1306. doi:10.1016/j.jhep.2010.11.008 21145851

[3] Myers RP, Shaheen AAM, Aspinall AI, Quinn RR, Burak KW. Gender, renal function, and outcomes on the liver transplant waiting list: assessment of revised MELD including estimated glomerular filtration rate. J Hepatol. 2011;54(3):462-470. doi:10.1016/j.jhep.2010.07.015 21109324

[4] Lai JC, Terrault NA, Vittinghoff E, Biggins SW. Height contributes to the gender difference in wait-list mortality under the MELD-based liver allocation system. Am J Transplant. 2010;10(12):2658-2664. doi:10.1111/j.1600-6143.2010.03326.x 21087414 PMC3059496

[5] Kim WR, Biggins SW, Kremers WK, . Hyponatremia and mortality among patients on the liver-transplant waiting list. N Engl J Med. 2008;359(10):1018-1026. doi:10.1056/NEJMoa0801209 18768945 PMC4374557

[6] Ruf AE, Kremers WK, Chavez LL, Descalzi VI, Podesta LG, Villamil FG. Addition of serum sodium into the MELD score predicts waiting list mortality better than MELD alone. Liver Transpl. 2005;11(3):336-343. doi:10.1002/lt.20329 15719386

[7] Mathur AK, Schaubel DE, Gong Q, Guidinger MK, Merion RM. Sex-based disparities in liver transplant rates in the United States. Am J Transplant. 2011;11(7):1435-1443. doi:10.1111/j.1600-6143.2011.03498.x 21718440 PMC3132137

[8] Moylan CA, Brady CW, Johnson JL, Smith AD, Tuttle-Newhall JE, Muir AJ. Disparities in liver transplantation before and after introduction of the MELD score. JAMA. 2008;300(20):2371-2378. doi:10.1001/jama.2008.720 19033587 PMC3640479

[9] Bryce CL, Angus DC, Arnold RM, . Sociodemographic differences in early access to liver transplantation services. Am J Transplant. 2009;9(9):2092-2101. doi:10.1111/j.1600-6143.2009.02737.x 19645706 PMC2880404

[10] Cullaro G, Sarkar M, Lai JC. Sex-based disparities in delisting for being “too sick” for liver transplantation. Am J Transplant. 2018;18(5):1214-1219. doi:10.1111/ajt.14608 29194969 PMC5910224

[11] Sarkar M, Watt KD, Terrault N, Berenguer M. Outcomes in liver transplantation: does sex matter? J Hepatol. 2015;62(4):946-955. doi:10.1016/j.jhep.2014.11.023 25433162 PMC5935797

[12] Locke JE, Shelton BA, Olthoff KM, . Quantifying sex-based disparities in liver allocation. JAMA Surg. 2020;155(7):e201129. doi:10.1001/jamasurg.2020.1129 32432699 PMC7240642

[13] Nephew LD, Goldberg DS, Lewis JD, Abt P, Bryan M, Forde KA. Exception points and body size contribute to gender disparity in liver transplantation. Clin Gastroenterol Hepatol. 2017;15(8):1286-1293.e2. doi:10.1016/j.cgh.2017.02.033 28288834 PMC10423635

[14] Mindikoglu AL, Emre SH, Magder LS. Impact of estimated liver volume and liver weight on gender disparity in liver transplantation. Liver Transpl. 2013;19(1):89-95. 23008117 10.1002/lt.23553 PMC3535518

[15] Allen AM, Heimbach JK, Larson JJ, . Reduced access to liver transplantation in women: role of height, MELD exception scores, and renal function underestimation. Transplantation. 2018;102(10):1710-1716. doi:10.1097/TP.0000000000002196 29620614 PMC6153066

[16] Mindikoglu AL, Regev A, Seliger SL, Magder LS. Gender disparity in liver transplant waiting-list mortality: the importance of kidney function. Liver Transpl. 2010;16(10):1147-1157. doi:10.1002/lt.22121 20879013 PMC3119710

[17] Cholongitas E, Marelli L, Kerry A, . Female liver transplant recipients with the same GFR as male recipients have lower MELD scores: a systematic bias. Am J Transplant. 2007;7(3):685-692. doi:10.1111/j.1600-6143.2007.01666.x 17217437

[18] Huo SC, Huo TI, Lin HC, . Is the corrected-creatinine model for end-stage liver disease a feasible strategy to adjust gender difference in organ allocation for liver transplantation? Transplantation. 2007;84(11):1406-1412. doi:10.1097/01.tp.0000282867.92367.d0 18091516

[19] Denny JC, Rutter JL, Goldstein DB, ; All of Us Research Program Investigators. The ‘All of Us’ research program. N Engl J Med. 2019;381(7):668-676. doi:10.1056/NEJMsr1809937 31412182 PMC8291101

[20] Ho DE, Imai K, King G, Stuart EA. MatchIt: nonparametric preprocessing for parametric causal inference. J Stat Softw. 2011. doi:10.18637/jss.v042.i08

[21] Thompson D, Waisanen L, Wolfe R, Merion RM, McCullough K, Rodgers A. Simulating the allocation of organs for transplantation. Health Care Manag Sci. 2004;7(4):331-338. doi:10.1007/s10729-004-7541-3 15717817

[22] Darden M, Parker G, Anderson E, Buell JF. Persistent sex disparity in liver transplantation rates. Surgery. 2021;169(3):694-699. doi:10.1016/j.surg.2020.06.028 32782116

[23] Wood NL, VanDerwerken D, Segev DL, Gentry SE. Correcting the sex disparity in MELD-Na. Am J Transplant. 2021;21(10):3296-3304. doi:10.1111/ajt.16731 34174151 PMC8500920

[24] Peralta CA, Risch N, Lin F, . The association of African ancestry and elevated creatinine in the coronary artery risk development in young adults (CARDIA) study. Am J Nephrol. 2010;31(3):202-208. doi:10.1159/000268955 20029176 PMC3487144

[25] Hsu J, Johansen KL, Hsu C-Y, Kaysen GA, Chertow GM. Higher serum creatinine concentrations in Black patients with chronic kidney disease: beyond nutritional status and body composition. Clin J Am Soc Nephrol. 2008;3(4):992-997. doi:10.2215/CJN.00090108 18417750 PMC2440282

[26] Borrell LN, Elhawary JR, Fuentes-Afflick E, . Race and genetic ancestry in medicine: a time for reckoning with racism. N Engl J Med. 2021;384(5):474-480. doi:10.1056/NEJMms2029562 33406325 PMC8979367

[27] Powe NR. Black kidney function matters: use or misuse of race? JAMA. 2020;324(8):737-738. doi:10.1001/jama.2020.13378 32761164

[28] Hsu CY, Yang W, Parikh RV, ; CRIC Study Investigators. Race, genetic ancestry, and estimating kidney function in CKD. N Engl J Med. 2021;385(19):1750-1760. doi:10.1056/NEJMoa2103753 34554660 PMC8994696

[29] Udler MS, Nadkarni GN, Belbin G, . Effect of genetic African ancestry on EGFR and kidney disease. J Am Soc Nephrol. 2015;26(7):1682-1692. doi:10.1681/ASN.2014050474 25349204 PMC4483587

