# Supplementary material for: Proposing a Sex-Adjusted Sodium-Adjusted MELD Score for Liver Transplant Allocation
Source: JAMA Surg. 2022 May 18;157(7):618–26. doi: 10.1001/jamasurg.2022.1548 (PMC9118088; doi:10.1001/jamasurg.2022.1548)
Supplement: Supplement 1. — eAppendix. eTable 1. Sample characteristics of Vanderbilt University Medical Center Synthetic Derivative (VUMC) and All of Us eTable 2. Descriptive statistics of median MELDNa component labs and median calculated MELDNa stratified by sex and liver status in VUMC eTable 3. Decompensation phenotype ICD9 and ICD10 codes eTable 4. Number of individuals with decompensation traits stratified by sex and liver status eTable 5. Descriptive statistics of MELDNa component labs in the All of Us Research Program eTable 6. Sex differences in median MELDNa component labs and calculated MELDNa stratified by liver status in VUMC eTable 7. Sex differences in median MELDNa component labs and calculated MELDNa stratified by liver status in All of Us eTable 8. Sex differences in maximum MELDNa component labs and calculated MELDNa stratified by liver status in VUMC eTable 9. Sex differences in average decompensation counts stratified by sex and liver status eFigure 1. Sex differences in maximum a) creatinine, b) INR, c) bilirubin, d) sodium, and e) calculated MELDNa stratified by liver status in VUMC eFigure 2. Sex differences in decompensation counts stratified by liver status in VUMC and All of Us eFigure 3. Decompensation counts by median calculated MELDNa stratified by sex and liver status [file jamasurg-e221548-s001.pdf]

Supplemental Online Content

Sealock JM, Ziogas IA, Zhao Z, et al. Proposing a sex-adjusted sodium-adjusted meld score for liver transplant allocation. *JAMA Surg.* Published online May 18, 2022. doi:10.1001/jamasurg.2022.1548

eAppendix.
eTable 1. Sample characteristics of Vanderbilt University Medical Center Synthetic Derivative (VUMC) and All of Us

eTable 2. Descriptive statistics of median MELDNa component labs and median calculated MELDNa stratified by sex and liver status in VUMC

eTable 3. Decompensation phenotype ICD9 and ICD10 codes

eTable 4. Number of individuals with decompensation traits stratified by sex and liver status

eTable 5. Descriptive statistics of MELDNa component labs in the All of Us Research Program

eTable 6. Sex differences in median MELDNa component labs and calculated MELDNa stratified by liver status in VUMC

eTable 7. Sex differences in median MELDNa component labs and calculated MELDNa stratified by liver status in All of Us

eTable 8. Sex differences in maximum MELDNa component labs and calculated MELDNa stratified by liver status in VUMC

eTable 9. Sex differences in average decompensation counts stratified by sex and liver status

eFigure 1. Sex differences in maximum a) creatinine, b) INR, c) bilirubin, d) sodium, and e) calculated MELDNa stratified by liver status in VUMC

eFigure 2. Sex differences in decompensation counts stratified by liver status in VUMC and All of Us

eFigure 3. Decompensation counts by median calculated MELDNa stratified by sex and liver status

This supplemental material has been provided by the authors to give readers additional information about their work.

© 2022 Sealock JM et al. *JAMA Surgery.*

eAppendix.

Calculation of OPTN MELDNa Scores

Calculation of OPTN MELDNa scores followed current clinical guidelines. Creatinine, INR, and bilirubin values were set to a minimum of 1 and creatinine levels were set to a maximum of 4. Sodium levels were restricted to a minimum of 125 and a maximum of 137. Calculated OPTN MELDNa scores were restricted to a maximum of 40. OPTN MELDNa was then calculated using the OPTN formula[20]:

$$MELD = 3.78*ln(bilirubin) + 11.2*ln(INR) + 9.57*ln(creatinine) + 6.43$$
$$MELDNa = MELD + 1.32*(137 - sodium) - [0.033*MELD*(137 - sodium)]$$

Decompensation Count by MELDNa Score

To determine how OPTN MELDNa scores track with the number of decompensation traits among males and females, linear regression models were fit between OPTN $MELDNa_{median}$ scores and decompensation counts. Analyses were stratified by liver status groups and sex (Supplementary Figure 2).

Replication in All of Us Research Program

Methods

All of Us contains data on over 271,000 individuals of whom 56,715 had recorded lab values for component MELDNa labs and were included in the replication analyses (eTable 1). Definitions for controls, liver disease, liver transplant, and decompensation traits were the same as in the VUMC sample. Lab values were filtered as described in VUMC and then used to calculate OPTN MELDNa scores (eTables 4-5). Sex differences in median lab values, median calculated MELDNa scores, and decompensation counts were assessed across the entire sample and within liver status groups using t-tests and Wilcoxon Rank Sum tests (eTable 7 & 9). Sex differences in lab values and MELDNa scores were also assessed with ANCOVA controlled for decompensation count (eTable 7).

Results

In All of Us controls, males had higher median creatinine (p=1.22x10^{-316}), INR (p=3.49x10^{-31}), and bilirubin levels (p=4.44x10^{-168}), but not sodium levels (p=0.643). Males had significantly higher median levels of all MELDNa labs within liver disease cases ($p_{creatinine}$=8.39x10^{-34}; p_{INR}=1.43x10^{-9}; $p_{bilirubin}$=8.64x10^{-13}; p_{sodium}=3.16x10^{-9}). Within liver transplant recipients, median lab values were not significantly different between males and females ($p_{creatinine}$=0.96; p_{INR}=0.53; $p_{bilirubin}$=0.32; p_{sodium}=0.57), however, it should be noted that sample size was small for this group (N<20; eTable 6, eFigure 3). In the comparison of median calculated MELDNa scores, males had significantly higher scores compared to females in controls (p=4.64x10^{-33}) and liver disease cases (p=1.87x10^{-14}).

Males had higher counts of decompensation traits within controls (p=5.76x10^{-6}) and within liver disease cases (p=3.15x10^{-6}), but not within liver transplant recipients (p=0.71) (eTable 8, eFigure 4).

© 2022 Sealock JM et al. *JAMA Surgery.*

eFigure 1. Sex differences in maximum a) creatinine, b) INR, c) bilirubin, d) sodium, and e) calculated MELDNa stratified by liver status in VUMC. In liver disease cases and controls, maximum values across the entire medical record were selected. In liver transplant cases, maximum before transplant was selected. Statistical significance was determined with Student's t-tests.

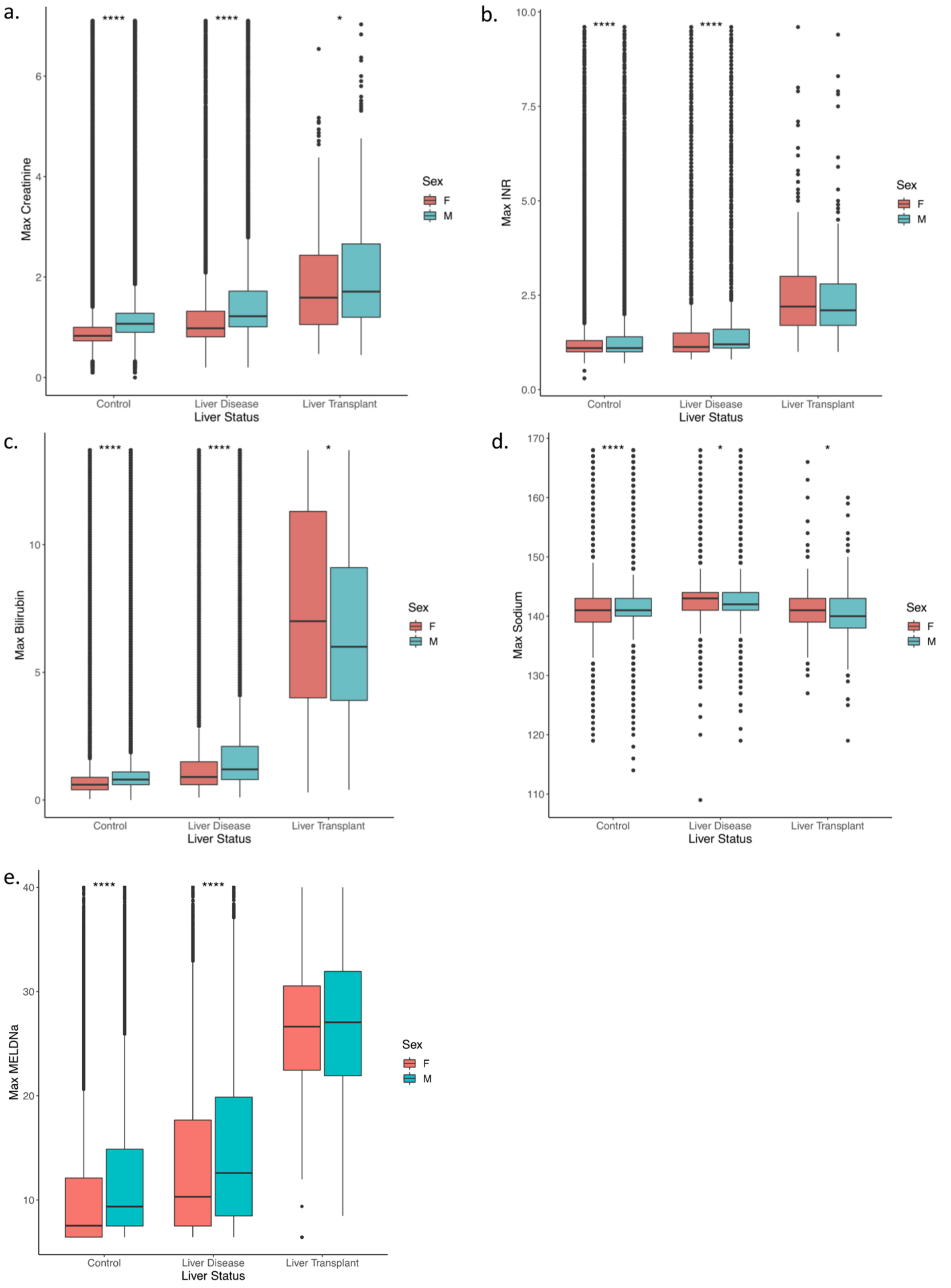

© 2022 Sealock JM et al. *JAMA Surgery.*

eFigure 2. Sex differences in decompensation counts stratified by liver status in VUMC and All of Us. For liver transplant recipients, decompensation traits prior to liver transplant date were utilized. For controls and liver disease cases, decompensation traits across the entire EHR were used. Sex differences were assessed using a t-test. ** denotes p<=0.01; ***: p<=0.001; ****: p<=0.0001.

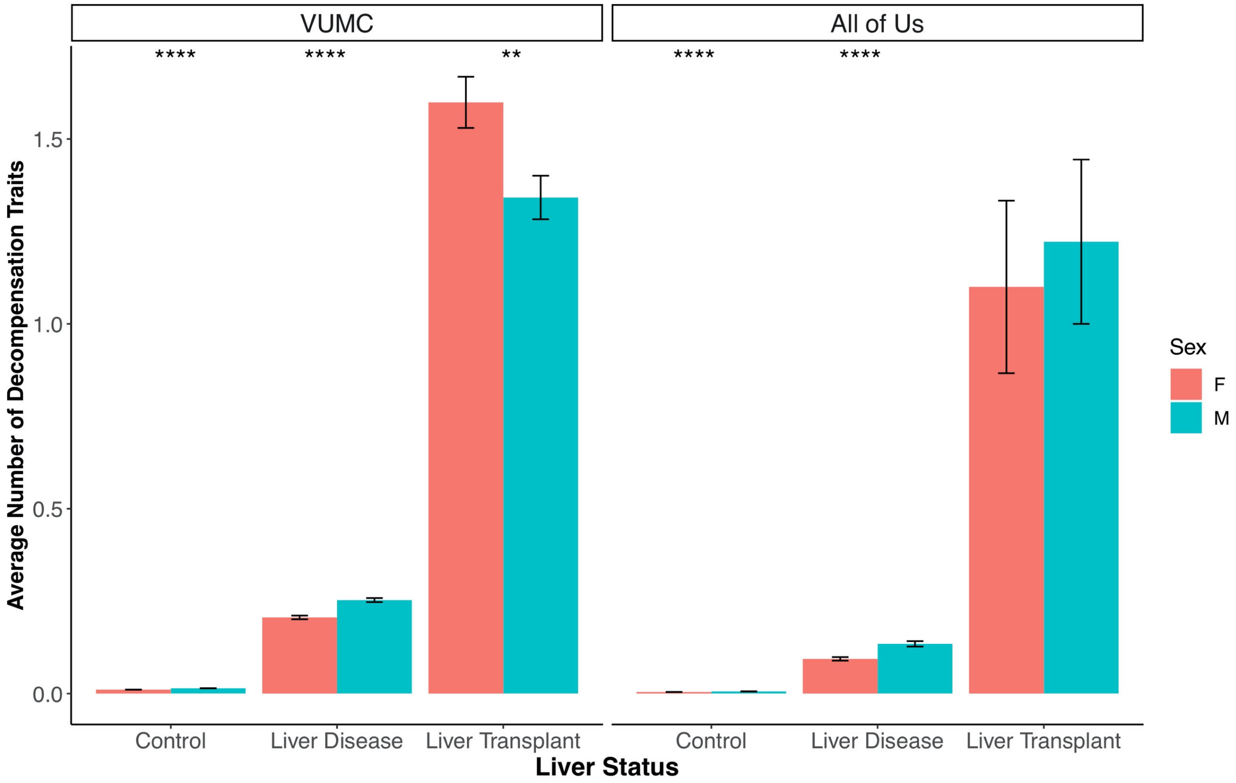

© 2022 Sealock JM et al. *JAMA Surgery.*

eFigure 3. Decompensation counts by median calculated MELDNa stratified by sex and liver status. A linear regression was fit between the number of decompensation traits and median MELDNa scores.

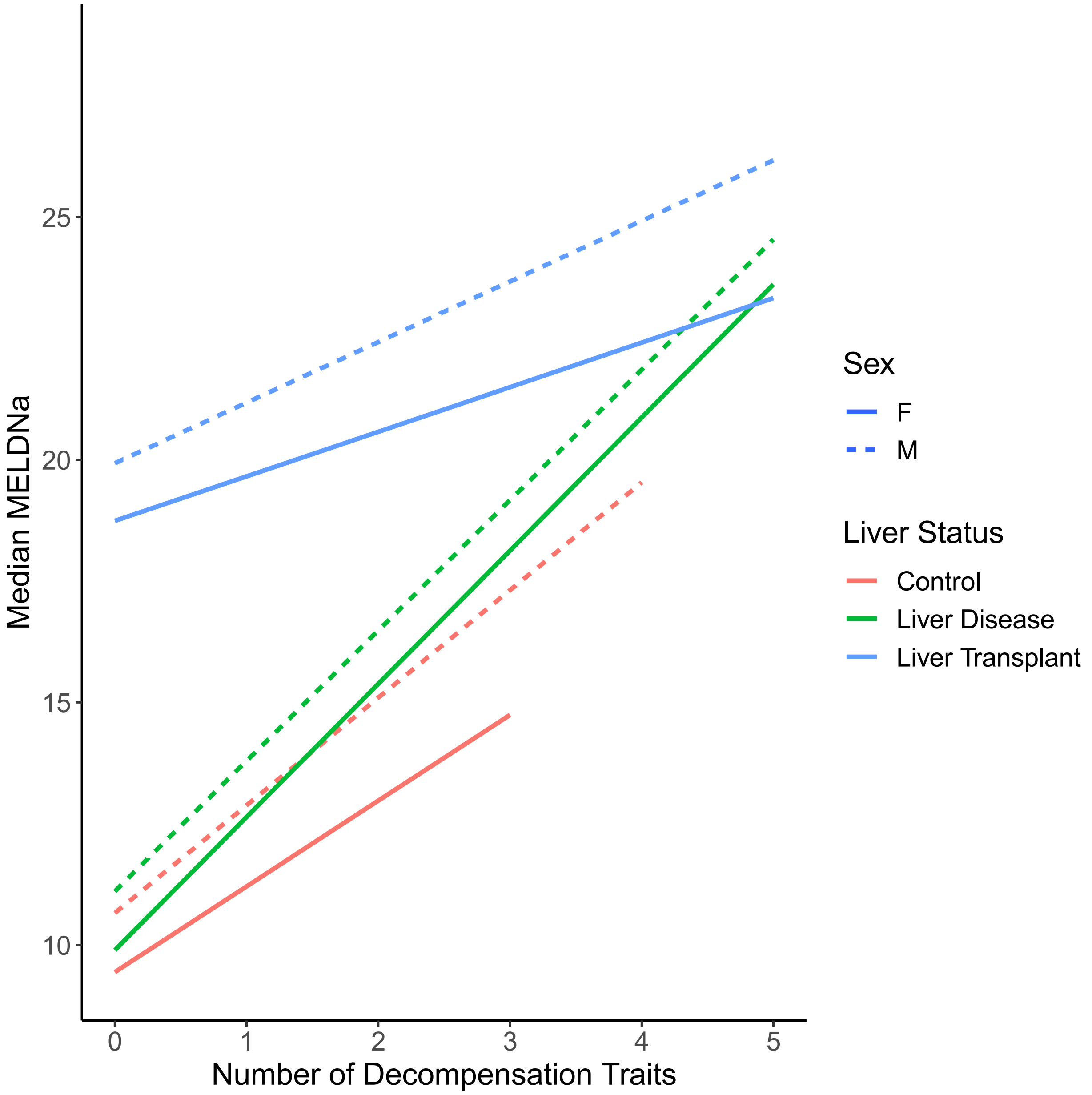

© 2022 Sealock JM et al. *JAMA Surgery.*

eTable 1. Sample characteristics of Vanderbilt University Medical Center Synthetic Derivative (VUMC) and All of Us.

	**Sample Source**	**All**	**Males**	**Females**
**N**	VUMC	623,931	263,955	359,976
	All of Us	56,715	19,715	36,154
**Liver Transplant Cases**	VUMC	601 (0.01%)	354 (0.13%)	247 (0.07%)
	All of Us	25 (0.44%)	<=20	<=20
**Liver Disease Cases**	VUMC	24,921 (4.0%)	12,247 (4.6%)	12,674 (3.5%)
	All of Us	6,422 (11.3%)	2,756	3,666
**Controls**	VUMC	598,409 (95.9%)	251,354 (95.2%)	347,055 (96.4%)
	All of Us	49,422 (87.1%)	16,944	32,478
**Median age (IQR)**	VUMC	44 (23 – 61)	45 (19 – 62)	43 (25 – 60)
	All of Us	58 (43 - 68)	60 (47 - 69)	57 (42 - 67)
**% White**	VUMC	81.40%	82.50%	80.60%
	All of Us	53.65%	55.73%	52.85%

© 2022 Sealock JM et al. *JAMA Surgery.*

eTable 2. Descriptive statistics of median MELDNa component labs and median calculated MELDNa stratified by sex and liver status in VUMC. For liver disease cases and controls, median was calculated across the entire medical record. In liver transplant cases, median was calculated before the liver transplant date.

Lab	Liver Status	Sex	N	Median	Mean	SD	Range	IQR
Creatinine	All	All	414,374	0.82	0.88	0.36	0 - 7.1	0.7 - 0.98
		Female	237,338	0.75	0.79	0.3	0.1 - 7.09	0.66 - 0.85
		Male	177,036	0.94	0.99	0.39	0 - 7.1	0.81 - 1.1
	Control	All	389,459	0.81	0.87	0.35	0 - 7.09	0.7 - 0.98
		Female	224,706	0.75	0.79	0.29	0.1 - 7.09	0.66 - 0.85
		Male	164,753	0.94	0.99	0.39	0 - 7.09	0.81 - 1.1
	Liver Disease	All	24,339	0.83	0.92	0.43	0.2 - 7.1	0.71 - 1
		Female	12,385	0.76	0.83	0.36	0.2 - 7.08	0.67 - 0.88
		Male	11,954	0.93	1.02	0.46	0.2 - 7.1	0.8 - 1.1
	Liver Transplant	All	576	1.06	1.19	0.56	0.27 - 4.63	0.8 - 1.42
		Female	247	0.93	1.11	0.57	0.27 - 4.63	0.71 - 1.3
		Male	329	1.14	1.26	0.55	0.35 - 4.16	0.85 - 1.49
INR	All	All	163,161	1.1	1.22	0.41	0.3 - 9.1	1 - 1.2
		Female	84,502	1.1	1.20	0.4	0.3 - 8.9	1 - 1.2
		Male	78,659	1.1	1.24	0.42	0.7 - 9.1	1 - 1.25
	Control	All	142,701	1.1	1.22	0.41	0.3 - 9.1	1 - 1.2
		Female	74,404	1.05	1.19	0.4	0.3 - 8.9	1 - 1.2
		Male	68,297	1.1	1.24	0.42	0.7 - 9.1	1 - 1.25
	Liver Disease	All	19,884	1.1	1.22	0.37	0.8 - 8.1	1 - 1.25
		Female	9,851	1.1	1.20	0.36	0.8 - 8.1	1 - 1.2
		Male	10,033	1.1	1.24	0.37	0.8 - 5	1 - 1.3
	Liver Transplant	All	576	1.6	1.79	0.65	0.9 - 7	1.4 - 1.95
		Female	247	1.6	1.80	0.73	0.9 - 7	1.34 - 2
		Male	329	1.65	1.78	0.59	1 - 6.2	1.4 - 1.9
Bilirubin	All	All	338,641	0.5	0.65	0.73	0 - 13.7	0.4 - 0.7
		Female	194,778	0.5	0.58	0.64	0 - 13.7	0.35 - 0.62
		Male	143,863	0.6	0.76	0.83	0 - 13.7	0.45 - 0.8
	Control	All	313,985	0.5	0.63	0.67	0 - 13.7	0.4 - 0.7
		Female	182,258	0.5	0.56	0.59	0 - 13.7	0.35 - 0.6
		Male	131,727	0.6	0.73	0.77	0 - 13.7	0.45 - 0.8
	Liver Disease	All	24,116	0.6	0.88	1.06	0.1 - 13.45	0.4 - 0.9
		Female	12,295	0.5	0.78	1.00	0.1 - 13.45	0.4 - 0.8
		Male	11,821	0.7	0.99	1.11	0.1 - 13.4	0.5 - 1
	Liver Transplant	All	540	3.3	4.11	2.76	0.25 - 13.6	2.1 - 5.32

© 2022 Sealock JM et al. *JAMA Surgery.*

Lab	Liver Status	Sex	N	Median	Mean	SD	Range	IQR
		Female	225	3.40	4.27	3.01	0.25 - 13.6	2 - 6.05
		Male	315	3.25	4.00	2.57	0.35 - 13.4	2.2 - 5.05
Sodium	All	All	408,746	139.00	139.02	2.31	109 - 161.5	138 - 140.5
		Female	232,661	139.00	139.03	2.28	109 - 160	138 - 140
		Male	176,085	139.00	139.00	2.36	114 - 161.5	138 - 140.5
	Control	All	383,855	139.00	139.06	2.29	114 - 161.5	138 - 140.5
		Female	220,055	139.00	139.06	2.26	117.5 - 160	138 - 140.5
		Male	163,800	139.00	139.06	2.32	114 - 161.5	138 - 140.5
	Liver Disease	All	24,315	139.00	138.5.0	2.47	109 - 154.5	137 - 140
		Female	12,359	139.00	138.65	2.39	109 - 151.5	137.5 - 140
		Male	11,956	139.00	138.34	2.54	116 - 154.5	137 - 140
	Liver Transplant	All	576	135.00	134.60	4.54	118 - 157	132 - 138
		Female	247	135.00	134.80	4.34	122 - 157	132 - 138
		Male	329	135.00	134.46	4.69	118 - 151	132 - 138
MELDNa	All	All	98,015	8.47	10.43	4.99	6.43 - 40	6.66 - 12.37
		Female	49,795	7.5.0	9.78	4.70	6.43 - 40	6.43 - 11.11
		Male	48,220	9.07	11.09	5.19	6.43 - 40	7.5 - 13.41
	Control	All	80,354	8.21	10.16	4.73	6.43 - 40	6.43 - 11.85
		Female	41,152	7.50	9.54	4.44	6.43 - 39.58	6.43 - 10.72
		Male	39,202	8.83	10.8	4.93	6.43 - 40	7.43 - 12.9
	Liver Disease	All	17,122	9.27	11.36	5.63	6.43 - 40	7.43 - 13.91
		Female	8,418	8.47	10.68	5.40	6.43 - 40	6.43 - 12.75
		Male	8,704	9.98	12.01	5.77	6.43 - 40	7.5 - 14.86
	Liver Transplant	All	539	20.86	21.09	6.17	6.43 - 39.82	16.35 - 25.62
		Female	225	20.09	20.21	6.15	6.43 - 33.63	15.8 - 24.54
		Male	314	21.62	21.72	6.11	6.43 - 39.82	16.96 - 26.2

© 2022 Sealock JM et al. *JAMA Surgery.*

eTable 3. Decompensation phenotype ICD9 and ICD10 codes.

ICD code	Description
578.0	Hematemesis
578.9	Hemorrhage of gastrointestinal tract, unspecified
789.5	Ascites
789.51	Malignant ascites
789.59	Other ascites
782.4	Jaundice, unspecified, not of newborn
572.2	Hepatic encephalopathy
K92.0	Hematemesis
K92.2	Gastrointestinal hemorrhage, unspecified
K70.11	Alcoholic hepatitis with ascites
K70.31	Alcoholic cirrhosis of liver with ascites
R17	Unspecified jaundice
K72.9	Hepatic failure, unspecified
K72.90	Hepatic failure, unspecified without coma
K72.91	Hepatic failure, unspecified with coma

© 2022 Sealock JM et al. *JAMA Surgery.*

eTable 4. Number of individuals with decompensation traits stratified by sex and liver status.

	Sample	N decompensated (%)	N males decompensated (%)	N females decompensated (%)
**All**	VUMC	12,297 (1.97%)	6,284 (2.38%)	6,013 (1.67%)
	All of Us	249 (0.11%)	140 (0.17%)	109 (0.08%)
**Controls**	VUMC	7,346 (1.23%)	3,602 (1.43%)	3,744 (1.08%)
	All of Us	43 (0.02%)	22 (0.03%)	21 (0.02%)
**Liver Disease**	VUMC	4,465 (17.91%)	2,409 (19.67%)	2,056 (16.22%)
	All of Us	195 (2.09%)	110 (2.77%)	85 (1.59%)
**Liver Transplant**	VUMC	486 (80.87%)	273 (77.12%)	213 (86.23%)
	All of Us	<=20 (39.3%)	<=20 (44.44%)	<=20 (30.0%)

© 2022 Sealock JM et al. *JAMA Surgery.*

eTable 5. Descriptive statistics of MELDNa component labs in the All of Us Research Program.

Lab	Liver Status	Sex	N	Median	Mean	SD	Range	IQR
Creatinine	All	All	37,962	0.82	0.92	0.62	0.2 - 14.69	0.7 - 1
		Female	25,529	0.78	0.83	0.45	0.2 - 12.44	0.7 - 0.89
		Male	12,433	1	1.13	0.84	0.23 - 14.69	0.89 - 1.13
	Control	All	32,099	0.81	0.90	0.54	0.2 - 14.69	0.7 - 0.99
		Female	21,929	0.78	0.81	0.40	0.2 - 12.44	0.69 - 0.88
		Male	10,170	1	1.10	0.73	0.23 - 14.69	0.9 - 1.12
	Liver Disease	All	5,803	0.85	1.03	0.91	0.3 - 12.66	0.72 - 1
		Female	3,579	0.8	0.89	0.64	0.4 - 10.8	0.7 - 0.9
		Male	2,224	1	1.25	1.20	0.3 - 12.66	0.86 - 1.2
	Liver Transplant	All	60	1.01	1.57	1.54	0.5 - 7.8	0.85 - 1.4
		Female	21	0.94	1.48	1.46	0.5 - 7.02	0.73 - 1.5
		Male	39	1.02	1.62	1.59	0.6 - 7.8	0.88 - 1.36
INR	All	All	23,495	1.04	1.13	0.32	0.5 - 8.55	1 - 1.1
		Female	14,083	1	1.11	0.30	0.5 - 8.55	1 - 1.1
		Male	9,412	1.1	1.17	0.34	0.7 - 5.7	1 - 1.2
	Control	All	17,274	1.02	1.12	0.31	0.5 - 8.55	1 - 1.1
		Female	10,578	1	1.10	0.30	0.5 - 8.55	1 - 1.1
		Male	6,696	1.07	1.15	0.33	0.7 - 4.4	1 - 1.15
	Liver Disease	All	6,197	1.05	1.15	0.32	0.8 - 5.7	1 - 1.2
		Female	3,496	1.01	1.12	0.29	0.8 - 3.7	1 - 1.1
		Male	2,701	1.1	1.19	0.36	0.8 - 5.7	1 - 1.2
	Liver Transplant	All	24	1.42	1.75	1.27	0.97 - 7.1	1.17 - 1.7
		Female	<20	1.45	2.02	1.93	0.97 - 7.1	1.1 - 1.71
		Male	<20	1.4	1.58	0.67	1 - 3.7	1.2 - 1.65
Bilirubin	All	All	35,959	0.5	0.59	0.54	0 - 16	0.4 - 0.7
		Female	24,327	0.45	0.53	0.50	0 - 16	0.35 - 0.6
		Male	11,632	0.6	0.71	0.60	0 - 15.25	0.45 - 0.8
	Control	All	30,222	0.5	0.57	0.47	0 - 16	0.4 - 0.7
		Female	20,803	0.45	0.52	0.45	0 - 16	0.35 - 0.6
		Male	9,419	0.6	0.68	0.49	0 - 12.6	0.45 - 0.8
	Liver Disease	All	5,680	0.5	0.66	0.74	0.1 - 15.25	0.4 - 0.7
		Female	3,505	0.5	0.58	0.64	0.1 - 14.55	0.4 - 0.6
		Male	2,175	0.6	0.79	0.86	0.1 - 15.25	0.5 - 0.8
	Liver Transplant	All	57	2.2	2.89	2.25	0.35 - 10.7	1.4 - 3.35

© 2022 Sealock JM et al. *JAMA Surgery.*

Lab	Liver Status	Sex	N	Median	Mean	SD	Range	IQR
		Female	<20	3.3	3.23	2.30	0.6 - 7.4	1.32 - 4.68
		Male	38	2.1	2.72	2.24	0.35 - 10.7	1.52 - 2.91
Sodium	All	All	39,976	139	138.78	2.27	122 - 151	137.5 - 140
		Female	26,663	139	138.81	2.22	122.5 - 151	137.5 - 140
		Male	13,313	139	138.72	2.37	122 - 150	137 - 140
	Control	All	33,612	139	138.83	2.27	122 - 151	137.5 - 140
		Female	22,820	139	138.83	2.23	122.5 - 151	137.5 - 140
		Male	10,792	139	138.83	2.35	122 - 150	137.5 - 140
	Liver Disease	All	6,317	139	138.53	2.25	123 - 147.5	137 - 140
		Female	3,829	139	138.72	2.15	123 - 146	137.5 - 140
		Male	2,488	138	138.25	2.35	124 - 147.5	137 - 140
	Liver Transplant	All	47	137	136.64	3.70	127 - 145	135 - 138.75
		Female	<20	136.5	137.04	3.46	131 - 145	135 - 138.88
		Male	33	137	136.47	3.83	127 - 144	134.5 - 138.5
MELDNa	All	All	6,535	8.41	10.06	4.48	6.43 - 37.77	6.96 - 11.38
		Female	4,153	7.54	9.36	3.99	6.43 - 32.25	6.43 - 10.22
		Male	2,382	9.38	11.26	5.00	6.43 - 37.77	7.5 - 13.43
	Control	All	4,439	8.09	9.69	4.16	6.43 - 33.73	6.79 - 10.76
		Female	2,924	7.5	9.17	3.81	6.43 - 31.62	6.43 - 9.85
		Male	1,515	9.07	10.71	4.60	6.43 - 33.73	7.5 - 12.17
	Liver Disease	All	2,083	8.66	10.77	4.96	6.43 - 37.77	7.39 - 12.89
		Female	1,225	8.09	9.82	4.37	6.43 - 32.25	6.54 - 10.97
		Male	858	10.31	12.14	5.42	6.43 - 37.77	7.76 - 15.1
	Liver Transplant	All	<20	17.31	18.35	7.08	10.12 - 31.01	12.01 - 22.37
		Female	<20	12.92	13.76	2.65	11.72 - 17.47	11.94 - 14.74
		Male	<20	20.79	20.39	7.57	10.12 - 31.01	16.76 - 25.39

© 2022 Sealock JM et al. *JAMA Surgery.*

eTable 6. Sex differences in median MELDNa component labs and calculated MELDNa stratified by liver status in VUMC. Differences were assessed using a Student's t-test, Wilcoxon rank sum tests, and ANCOVA tests controlled for decompensation count as a proxy for disease severity.

**Lab**	**Group**	**T-test p-value**	**Wilcoxon p-value**	**ANCOVA p-value**
Creatinine	All	$<2.22 \times 10^{-308}$	$<2.22 \times 10^{-308}$	$<2.22 \times 10^{-308}$
	Controls	$<2.22 \times 10^{-308}$	$<2.22 \times 10^{-308}$	$<2.22 \times 10^{-308}$
	Liver Disease	1.03×10^{-291}	$<2.22 \times 10^{-308}$	1.43×10^{-296}
	Liver Transplant	0.001	2.96×10^{-06}	8.66×10^{-4}
INR	All	1.47×10^{-128}	$<2.22 \times 10^{-308}$	2.85×10^{-130}
	Controls	2.65×10^{-114}	$<2.22 \times 10^{-308}$	4.12×10^{-115}
	Liver Disease	1.80×10^{-14}	1.81×10^{-36}	3.16×10^{-15}
	Liver Transplant	0.738	0.232	0.728
Bilirubin	All	$<2.22 \times 10^{-308}$	$<2.22 \times 10^{-308}$	$<2.22 \times 10^{-308}$
	Controls	$<2.22 \times 10^{-308}$	$<2.22 \times 10^{-308}$	$<2.22 \times 10^{-308}$
	Liver Disease	6.42×10^{-54}	3.16×10^{-276}	7.65 x 10-60
	Liver Transplant	0.262	0.793	0.249
Sodium	All	$<2.22 \times 10^{-308}$	0.754	1.28×10^{-04}
	Controls	0.599	1.76×10^{-4}	0.597
	Liver Disease	1.88×10^{-23}	9.55×10^{-27}	1.29×10^{-24}
	Liver Transplant	0.364	0.813	0.357
MELD-Na	All	$<2.22 \times 10^{-308}$	$<2.22 \times 10^{-308}$	$<2.22 \times 10^{-308}$
	Controls	3.16×10^{-313}	$<2.22 \times 10^{-308}$	8.67×10^{-319}
	Liver Disease	2.20×10^{-54}	4.16×10^{-101}	4.70×10^{-61}

© 2022 Sealock JM et al. *JAMA Surgery.*

eTable 7. Sex differences in median MELDNa component labs and calculated MELDNa stratified by liver status in All of Us. Differences were assessed using a Student's t-test, Wilcoxon rank sum tests, and ANCOVA tests controlled for decompensation count as a proxy for disease severity.

Lab	Group	T-test p-value	Wilcoxon p-value	ANCOVA p-value
Creatinine	All	$<2.22 \times 10^{-308}$	$<2.22 \times 10^{-308}$	$<2.22 \times 10^{-308}$
	Controls	1.22×10^{-316}	$<2.22 \times 10^{-308}$	$<2.22 \times 10^{-308}$
	Liver Disease	8.39×10^{-34}	6.07×10^{-259}	3.27×10^{-66}
	Liver Transplant	0.961	0.545	0.960
INR	All	8.48×10^{-40}	1.07×10^{-106}	3.12×10^{-42}
	Controls	3.49×10^{-31}	1.72×10^{-74}	1.25×10^{-28}
	Liver Disease	1.43×10^{-09}	4.88E-30	3.02×10^{-15}
	Liver Transplant	0.528	0.976	0.473
Bilirubin	All	5.11×10^{-169}	$<2.22 \times 10^{-308}$	8.58×10^{-193}
	Controls	4.44×10^{-168}	$<2.22 \times 10^{-308}$	1.97×10^{-170}
	Liver Disease	8.64×10^{-13}	1.30×10^{-100}	1.56×10^{-24}
	Liver Transplant	0.317	0.488	0.328
Sodium	All	4.08×10^{-03}	4.08×10^{-03}	3.26×10^{-03}
	Controls	0.6425	0.220	0.493
	Liver Disease	3.16×10^{-09}	3.04×10^{-17}	1.29×10^{-14}
	Liver Transplant	0.565	0.641	0.481
MELD-Na	All	3.31×10^{-48}	6.03×10^{-76}	7.63×10^{-47}
	Controls	4.64×10^{-33}	2.26×10^{-43}	5.01×10^{-24}
	Liver Disease	1.87×10^{-14}	1.36×10^{-31}	5.87×10^{-24}

© 2022 Sealock JM et al. *JAMA Surgery.*

eTable 8. Sex differences in maximum MELDNa component labs and calculated MELDNa stratified by liver status in VUMC. Statistical significance was assessed using a Student's t-test.

Lab	Group	p-value
Creatinine	All	$<2.22 \times 10^{-308}$
	Control	$<2.22 \times 10^{-308}$
	Liver Disease	5.19×10^{-125}
	Liver Transplant	0.047
INR	All	7.11×10^{-55}
	Control	4.17×10^{-49}
	Liver Disease	1.00×10^{-05}
	Liver Transplant	0.198
Bilirubin	All	$<2.22 \times 10^{-308}$
	Control	$<2.22 \times 10^{-308}$
	Liver Disease	3.71×10^{-40}
	Liver Transplant	0.026
Sodium	All	1.19×10^{-195}
	Control	2.41×10^{-177}
	Liver Disease	0.041
	Liver Transplant	0.021
MELDNa	All	8.51×10^{-304}
	Control	6.41×10^{-258}
	Liver Disease	2.33×10^{-46}
	Liver Transplant	0.186

© 2022 Sealock JM et al. *JAMA Surgery.*

eTable 9. Sex differences in average decompensation counts stratified by sex and liver status. Statistical significance was assessed using Student's t-tests and Wilcoxon rank sum tests.

**Liver Status**	**Sample**	**Female Average (SD)**	**Male Average (SD)**	**T-test p-value**	**Wilcoxon-p-value**
All	VUMC	0.019 (0.16)	0.027 (0.20)	2.20×10^{-73}	2.11×10^{-79}
	All of Us	0.008 (0.10)	0.012 (0.13)	1.31×10^{-16}	6.85×10^{-16}
Controls	VUMC	0.011 (0.11)	0.144 (0.13)	2.86×10^{-31}	5.79×10^{-31}
	All of Us	0.004 (0.07)	0.006 (0.08)	5.76×10^{-06}	4.65×10^{-06}
Liver Disease	VUMC	0.206 (0.56)	0.253 (0.61)	2.75×10^{-10}	2.83×10^{-11}
	All of Us	0.094 (0.36)	0.135 (0.46)	3.15×10^{-06}	2.76×10^{-05}
Liver Transplant	VUMC	1.599 (1.09)	1.341 (1.11)	4.81×10^{-3}	2.73×10^{-03}
	All of Us	1.100 (0.74)	1.222 (0.94)	0.708	0.742

© 2022 Sealock JM et al. *JAMA Surgery.*
