# Supplementary material for: Proposing a Sex-Adjusted Sodium-Adjusted MELD Score for Liver Transplant Allocation
Source: JAMA Surg. 2022 May 18;157(7):618–26. doi: 10.1001/jamasurg.2022.1548 (PMC9118088; doi:10.1001/jamasurg.2022.1548)
Supplement: Supplement 2. — ICD-9 and ICD-10 codes used to define liver disease cases [file jamasurg-e221548-s002.pdf]

Sealock JM, Ziogas IA, Zhao Z, et al. Proposing a sex-adjusted sodium-adjusted meld score for liver transplant allocation. *JAMA Surg.* Published online May 18, 2022. doi:10.1001/jamasurg.2022.1548

ICD-9 and ICD-10 codes used to define liver disease cases

ICD Code	Description
570	Acute and subacute necrosis of liver
571	Chronic liver disease and cirrhosis
571	Alcoholic fatty liver
571.1	Acute alcoholic hepatitis
571.2	Alcoholic cirrhosis of liver
571.3	Alcoholic liver damage, unspecified
571.4	Chronic hepatitis
571.4	Chronic hepatitis, unspecified
571.41	Chronic persistent hepatitis
571.42	Autoimmune hepatitis
571.49	Other chronic hepatitis
571.5	Cirrhosis of liver without mention of alcohol
571.6	Biliary cirrhosis
571.8	Other chronic nonalcoholic liver disease
571.9	Unspecified chronic liver disease without mention of alcohol
572	Liver abscess and sequelae of chronic liver disease
572	Abscess of liver
572.1	Portal pyemia
572.2	Hepatic encephalopathy
572.3	Portal hypertension
572.4	Hepatorenal syndrome
572.8	Other sequelae of chronic liver disease
573	Other disorders of liver
573	Chronic passive congestion of liver
573.1	Hepatitis in viral diseases classified elsewhere
573.2	Hepatitis in other infectious diseases classified elsewhere
573.3	Hepatitis, unspecified
573.4	Hepatic infarction
573.5	Hepatopulmonary syndrome
573.8	Other specified disorders of liver
573.9	Unspecified disorder of liver
70	Viral hepatitis
70	Viral hepatitis A with hepatic coma
70.1	Viral hepatitis A without mention of hepatic coma
70.2	Viral hepatitis b with hepatic coma
70.2	Viral hepatitis B with hepatic coma, acute or unspecified, without mention of hepatitis delta
70.21	Viral hepatitis B with hepatic coma, acute or unspecified, with hepatitis delta
70.22	Chronic viral hepatitis B with hepatic coma without hepatitis delta

© 2022 Sealock JM et al. *JAMA Surgery* .

ICD Code	Description
70.23	Chronic viral hepatitis B with hepatic coma with hepatitis delta
70.3	Viral hepatitis b without mention of hepatic coma
70.3	Viral hepatitis B without mention of hepatic coma, acute or unspecified, without mention of hepatitis delta
70.31	Viral hepatitis B without mention of hepatic coma, acute or unspecified, with hepatitis delta
70.32	Chronic viral hepatitis B without mention of hepatic coma without mention of hepatitis delta
70.33	Chronic viral hepatitis B without mention of hepatic coma with hepatitis delta
70.4	Other specified viral hepatitis with hepatic coma
70.41	Acute hepatitis C with hepatic coma
70.42	Hepatitis delta without mention of active hepatitis B disease with hepatic coma
70.43	Hepatitis E with hepatic coma
70.44	Chronic hepatitis C with hepatic coma
70.49	Other specified viral hepatitis with hepatic coma
70.5	Other specified viral hepatitis without mention of hepatic coma
70.51	Acute hepatitis C without mention of hepatic coma
70.52	Hepatitis delta without mention of active hepatitis B disease or hepatic coma
70.53	Hepatitis E without mention of hepatic coma
70.54	Chronic hepatitis C without mention of hepatic coma
70.59	Other specified viral hepatitis without mention of hepatic coma
70.6	Unspecified viral hepatitis with hepatic coma
70.7	Unspecified viral hepatitis c
70.7	Unspecified viral hepatitis C without hepatic coma
70.71	Unspecified viral hepatitis C with hepatic coma
70.9	Unspecified viral hepatitis without mention of hepatic coma
K70	Alcoholic liver disease
K70.0	Alcoholic fatty liver
K70.1	Alcoholic hepatitis
K70.10	Alcoholic hepatitis without ascites
K70.11	Alcoholic hepatitis with ascites
K70.2	Alcoholic fibrosis and sclerosis of liver
K70.3	Alcoholic cirrhosis of liver
K70.30	Alcoholic cirrhosis of liver without ascites
K70.31	Alcoholic cirrhosis of liver with ascites
K70.4	Alcoholic hepatic failure
K70.40	Alcoholic hepatic failure without coma
K70.41	Alcoholic hepatic failure with coma
K70.9	Alcoholic liver disease, unspecified

© 2022 Sealock JM et al. *JAMA Surgery* .

Sealock JM, Ziogas IA, Zhao Z, et al. Proposing a sex-adjusted sodium-adjusted meld score for liver transplant allocation. *JAMA Surg.* Published online May 18, 2022. doi:10.1001/jamasurg.2022.1548

ICD Code	Description
K71	Toxic liver disease
K71.0	Toxic liver disease with cholestasis
K71.1	Toxic liver disease with hepatic necrosis
K71.10	Toxic liver disease with hepatic necrosis, without coma
K71.11	Toxic liver disease with hepatic necrosis, with coma
K71.2	Toxic liver disease with acute hepatitis
K71.3	Toxic liver disease with chronic persistent hepatitis
K71.4	Toxic liver disease with chronic lobular hepatitis
K71.5	Toxic liver disease with chronic active hepatitis
K71.50	Toxic liver disease with chronic active hepatitis without ascites
K71.51	Toxic liver disease with chronic active hepatitis with ascites
K71.6	Toxic liver disease with hepatitis, not elsewhere classified
K71.7	Toxic liver disease with fibrosis and cirrhosis of liver
K71.8	Toxic liver disease with other disorders of liver
K71.9	Toxic liver disease, unspecified
K72	Hepatic failure, not elsewhere classified
K72.0	Acute and subacute hepatic failure
K72.00	Acute and subacute hepatic failure without coma
K72.01	Acute and subacute hepatic failure with coma
K72.1	Chronic hepatic failure
K72.10	Chronic hepatic failure without coma
K72.11	Chronic hepatic failure with coma
K72.9	Hepatic failure, unspecified
K72.90	Hepatic failure, unspecified without coma
K72.91	Hepatic failure, unspecified with coma
K73	Chronic hepatitis, not elsewhere classified
K73.0	Chronic persistent hepatitis, not elsewhere classified
K73.1	Chronic lobular hepatitis, not elsewhere classified
K73.2	Chronic active hepatitis, not elsewhere classified
K73.8	Other chronic hepatitis, not elsewhere classified
K73.9	Chronic hepatitis, unspecified
K74	Fibrosis and cirrhosis of liver
K74.0	Hepatic fibrosis
K74.1	Hepatic sclerosis
K74.2	Hepatic fibrosis with hepatic sclerosis
K74.3	Primary biliary cirrhosis
K74.4	Secondary biliary cirrhosis
K74.5	Biliary cirrhosis, unspecified
K74.6	Other and unspecified cirrhosis of liver
K74.60	Unspecified cirrhosis of liver

© 2022 Sealock JM et al. *JAMA Surgery* .

ICD Code	Description
K74.69	Other cirrhosis of liver
K75	Other inflammatory liver diseases
K75.0	Abscess of liver
K75.1	Phlebitis of portal vein
K75.2	Nonspecific reactive hepatitis
K75.3	Granulomatous hepatitis, not elsewhere classified
K75.4	Autoimmune hepatitis
K75.8	Other specified inflammatory liver diseases
K75.81	Nonalcoholic steatohepatitis (NASH)
K75.89	Other specified inflammatory liver diseases
K75.9	Inflammatory liver disease, unspecified
K76	Other diseases of liver
K76.0	Fatty (change of) liver, not elsewhere classified
K76.1	Chronic passive congestion of liver
K76.2	Central hemorrhagic necrosis of liver
K76.3	Infarction of liver
K76.4	Peliosis hepatis
K76.5	Hepatic veno-occlusive disease
K76.6	Portal hypertension
K76.7	Hepatorenal syndrome
K76.8	Other specified diseases of liver
K76.81	Hepatopulmonary syndrome
K76.89	Other specified diseases of liver
K76.9	Liver disease, unspecified
K77	Liver disorders in diseases classified elsewhere
B15	Acute hepatitis A
B15.0	Hepatitis A with hepatic coma
B15.9	Hepatitis A without hepatic coma
B16	Acute hepatitis B
B16.0	Acute hepatitis B with delta-agent with hepatic coma
B16.1	Acute hepatitis B with delta-agent without hepatic coma
B16.2	Acute hepatitis B without delta-agent with hepatic coma
B16.9	Acute hepatitis B without delta-agent and without hepatic coma
B17	Other acute viral hepatitis
B17.0	Acute delta-(super) infection of hepatitis B carrier
B17.1	Acute hepatitis C
B17.10	Acute hepatitis C without hepatic coma
B17.11	Acute hepatitis C with hepatic coma
B17.2	Acute hepatitis E
B17.8	Other specified acute viral hepatitis

© 2022 Sealock JM et al. *JAMA Surgery* .

Sealock JM, Ziogas IA, Zhao Z, et al. Proposing a sex-adjusted sodium-adjusted meld score for liver transplant allocation. *JAMA Surg.* Published online May 18, 2022. doi:10.1001/jamasurg.2022.1548

ICD Code	Description
B17.9	Acute viral hepatitis, unspecified
B18	Chronic viral hepatitis
B18.0	Chronic viral hepatitis B with delta-agent
B18.1	Chronic viral hepatitis B without delta-agent
B18.2	Chronic viral hepatitis C
B18.8	Other chronic viral hepatitis
B18.9	Chronic viral hepatitis, unspecified
B19	Unspecified viral hepatitis
B19.0	Unspecified viral hepatitis with hepatic coma
B19.1	Unspecified viral hepatitis B
B19.10	Unspecified viral hepatitis B without hepatic coma
B19.11	Unspecified viral hepatitis B with hepatic coma
B19.2	Unspecified viral hepatitis C
B19.20	Unspecified viral hepatitis C without hepatic coma
B19.21	Unspecified viral hepatitis C with hepatic coma
B19.9	Unspecified viral hepatitis without hepatic coma

© 2022 Sealock JM et al. *JAMA Surgery* .
